# Use of the Electronic Medical Record to Assess Pancreas Size in Type 1 Diabetes

**DOI:** 10.1371/journal.pone.0158825

**Published:** 2016-07-08

**Authors:** John Virostko, Melissa Hilmes, Kelsey Eitel, Daniel J. Moore, Alvin C. Powers

**Affiliations:** 1 Vanderbilt University Institute of Imaging Science, Vanderbilt University, Nashville, Tennessee, United States of America; 2 Department of Radiology and Radiological Sciences, Vanderbilt University, Nashville, Tennessee, United States of America; 3 Department of Pediatrics, Vanderbilt University, Nashville, Tennessee, United States of America; 4 Loyola University Chicago Stritch School of Medicine, Maywood, Illinois, United States of America; 5 Department of Pathology, Immunology, and Microbiology, Vanderbilt University, Nashville, Tennessee, United States of America; 6 Department of Medicine, Division of Diabetes, Endocrinology, and Metabolism, Vanderbilt University, Nashville, Tennessee, United States of America; 7 Department of Molecular Physiology and Biophysics, Vanderbilt University, Nashville, Tennessee, United States of America; 8 VA Tennessee Valley Healthcare System, Nashville, Tennessee, United States of America; University of Bremen, GERMANY

## Abstract

**Aims:**

This study harnessed the electronic medical record to assess pancreas volume in patients with type 1 diabetes (T1D) and matched controls to determine whether pancreas volume is altered in T1D and identify covariates that influence pancreas volume.

**Methods:**

This study included 25 patients with T1D and 25 age-, sex-, and weight-matched controls from the Vanderbilt University Medical Center enterprise data warehouse. Measurements of pancreas volume were made from medical imaging studies using magnetic resonance imaging (MRI) or computed tomography (CT).

**Results:**

Patients with T1D had a pancreas volume 47% smaller than matched controls (41.16 ml vs. 77.77 ml, P < 0.0001) as well as pancreas volume normalized by subject body weight, body mass index, or body surface area (all P < 0.0001). Pancreatic volume was smaller with a longer duration of T1D across the patient population (N = 25, P = 0.04). Additionally, four individual patients receiving multiple imaging scans displayed progressive declines in pancreas volume over time (~ 6% of volume/year), whereas five controls scanned a year apart did not exhibit a decline in pancreas size (P = 0.03). The pancreas was uniformly smaller on the right and left side of the abdomen.

**Conclusions:**

Pancreas volume declines with disease duration in patients with T1D, suggesting a protracted pathological process that may include the exocrine pancreas.

## Introduction

Type 1 diabetes (T1D) is preceded by a lengthy subclinical period during which the insulin-producing pancreatic beta cells are destroyed by an autoimmune process. This subclinical period is a promising window for early diagnosis and therapeutic intervention [1]. Beta cell mass cannot be quantified in vivo in humans, however, limiting our ability to identify and track patients in this window. Surrogate early markers of T1D are sought to aid disease prognostication, surveillance, and clinical management.

Reduced pancreas size and weight is a known clinical feature in patients with longstanding type 1 diabetes [2–5]. Surprisingly, recent magnetic resonance imaging (MRI) studies have demonstrated reduced pancreatic volume (by 26–31%) within months of diagnosis [6, 7], suggesting that the size of the pancreas may be reduced prior to disease onset. Supporting this finding, organ donor studies indicate that pancreas weight is decreased in individuals without diabetes who express serum autoantibodies that portend the development of T1D [8]. These results suggest that reduced pancreas volume may be an early biomarker for type 1 diabetes and raise fundamental questions that challenge our understanding of T1D pathogenesis including whether the diabetes-prone pancreas is intrinsically abnormal and to what degree endocrine-exocrine interactions contribute to pancreatic health.

Although it is known that weight [9] and age [10] affect pancreas volume, previous studies of pancreas volume in type 1 diabetes have not included controls individually matched for these parameters. Additionally, pancreas volume has not been studied concurrently in adult and pediatric patients with T1D, despite the high and increasing [11] incidence of T1D in children. Finally, longitudinal measurements have not been performed in the same patient over the course of T1D, limiting our understanding of the dynamics of pancreas volume. In this study we have leveraged the Vanderbilt University Medical Center electronic medical record to perform a matched case-control study of pancreas volume in type 1 diabetes, including both pediatric patients and patients with multiple longitudinal measurements.

## Materials and Methods

### Data Sources

This study was performed using the Research Derivative database available at Vanderbilt University. The Research Derivative is a database of clinical and related data derived from Vanderbilt University Medical Center’s clinical systems and restructured for research. Data is repurposed from Vanderbilt’s enterprise data warehouse, which includes data from StarPanel, Vanderbilt’s integrated web-based electronic health record portal, and health management systems including the Vanderbilt Perioperative Information Management System, the Operating Room Management Information System, EPIC, Medipac, and HEO. The medical record number and other person identifiers are preserved within the database. Data types include reimbursement codes, clinical notes and documentation, nursing records, medication data, laboratory data, encounter and visit data, among others. Output includes structured data points, such as International Classification of Diseases, 9th edition (ICD-9) codes and encounter dates, semi-structured data such as laboratory tests and results, and unstructured data such as physician progress reports [12]. Parameters derived from the patient record included sex, gravidity, age, height, weight, comorbidities, medication history, insulin usage, and glycated hemoglobin (HbA1c). The Vanderbilt Institutional Review Board granted this study an exemption from review under federal regulation 45 CFR 46.101(b)(4) for the use of existing data in such a manner that subjects cannot be identified.

### Study Population

**Fig 1:**
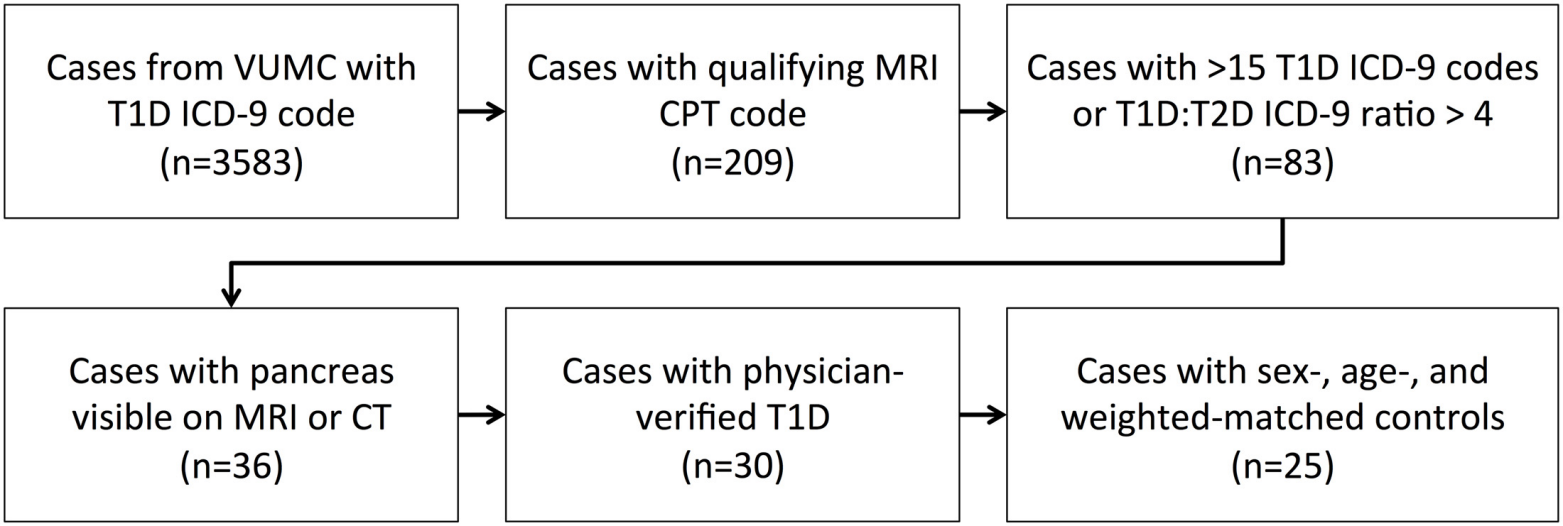
Flowchart demonstrating criteria used to identify patient records for inclusion in the study.

A flowchart demonstrating inclusion criteria for this study is shown in Fig 1. Study participants with type 1 diabetes were identified from a database of patients with outpatient visits to the Vanderbilt Diabetes Clinic who received a diagnosis of either 250.01 (controlled T1D) or 250.03 (uncontrolled T1D) using ICD-9 codes. Current Procedural Terminology (CPT) codes were then used to identify patients who had undergone magnetic resonance imaging (MRI) of the abdomen, chest, pelvis, or spine at Vanderbilt between 2000 and 2013. MRI scans used in this study were identified using CPT codes for abdominal MRI (74181, 74182, 74183), chest MRI (71550, 71551, 71552), pelvis MRI (72195, 72196, 72197), lumbar spine MRI (72148, 72158), thoracic spine MRI (72146, 72157), abdomen angiography (74185), chest angiography (71555), pelvis angiography (72198), and spine angiography (72159). An endocrinologist performed a preliminary review of the medical records of cases with qualifying CPT codes and identified contamination from patients with type 2 diabetes. To limit this contamination, stricter inclusion criteria were enforced for diagnosis of T1D. Instead of one occurrence of an ICD-9 for type 1 diabetes, cases were filtered to include only records with either >15 occurrences of the type 1 diabetes ICD-9 codes or a ratio of type 1 diabetes ICD-9 codes to type 2 diabetes ICD-9 codes greater than four. MRI images for each qualifying patient were viewed using the Vanderbilt picture archiving and communication system. As the MRI was not taken for the purpose of viewing pancreas pathology, not every imaging study included the whole pancreas. However, many of the cases identified for this study through qualifying MRI procedures also received abdominal computed tomography (CT). In cases where the entire pancreas was not visible by MRI, computed tomography (CT) images of the abdomen were analyzed, when available. To confirm type 1 diabetes diagnosis, the medical records were examined by a board-certified endocrinologist who examined lab reports, including c-peptide measurements, fasting glucose measurements, and autoantibody levels, as well as medication reports including insulin usage and made a diagnosis based on the ADA standard definition of type 1 diabetes. Controls matched to the patient population by sex, age, and weight were identified using the Vanderbilt Research Derivative database and screened to exclude pancreas pathology (cystic fibrosis, pancreatic masses). The maximum allowable difference in weight and age between controls and cases was 30%.

For longitudinal measurements made multiple times in the same control, pancreas volume was measured as part of a prospective study at Vanderbilt University. For these five control subjects, MRI measurements were made at 1 year increments. All measurements were made identically to those performed on retrospective data sets.

### Pancreas Volume

Pancreas volume was calculated from MRI or CT images stored in the Vanderbilt picture archiving and communication system. The pancreas was manually outlined on axial image slices using IMPAX 6 software (Afga Healthcare, Mortsel, Belgium) by a board-certified attending radiologist (M.H.). The areas of each slice were summed throughout the volume of the pancreas. Pancreas volume was calculated by multiplying the total pancreas area by the slice thickness (including the slice gap for MRI images) [13, 14]. The reading radiologist was not blinded to the medical record of the patient so that confounding pathologies could be identified and verified when reading the medical image. However, the reading radiologist did not calculate the pancreas volume from the outlined pancreas slices, to limit potential measurement bias.

To determine regional differences in pancreas volume the abdomen was bisected into left and right halves using a line drawn from spine to navel. Pancreas areas were split into left and right halves using this midsagittal plane. Pancreas volume for each half of the abdomen was calculated as described above. The pancreas volume to the left of the spine incorporated the pancreas head and portions of the pancreas body while the pancreas volume to the right of the spine included the pancreas tail and portions of the pancreas body.

### Statistical Analysis

Statistical analysis was performed using SPSS software, version 22 (IBM, Armonk, NY). Analysis of nominal data was made using Fisher’s Exact test. Pairwise comparisons of interval data were made using the Wilcoxon Signed-Rank Test. Univariate associations between interval variables were calculated using Pearson’s correlation. Multivariable associations were calculated using multiple linear regression. Data are presented in the text as either mean plus or minus standard deviation or median with interquartile range, as designated in the text. A significance level of p < 0.05 was deemed statistically significant in all analyses. When appropriate, Bonferroni correction was used to adjust for multiple comparisons.

## Results

### Subject Characteristics

A total of 25 patients with type 1 diabetes and 25 sex-, age-, and weight-matched controls were analyzed in this study. Patients and controls were matched 1:1 for sex and well matched for age, weight, height, body mass index (BMI), and body surface area (BSA, as calculated by the Du Bois method [15]) (Table 1). Among imaging techniques evaluated for this study, patients with T1D had a greater number of MRIs analyzed (6) than matched controls (1). Medical indications responsible for the imaging scan were well matched, with the exception that pancreatitis was more common in patients with diabetes (4 patients vs. 0 controls) and Crohn’s disease was more common in controls (3 controls vs. 0 patients).

**Table 1:** Demographic and Imaging Characteristics for Patients and Controls in This Study.

	Type 1 Diabetes (n = 25)	Controls (n = 25)	P value
Men, No. (%)	10 (40%)	10 (40%)	1.00^a^
Age, median (IQR), y	27.85 (16.15–41.46)	28.12 (16.55–41.88)	0.16^a^
Weight, median (IQR), kg	72.57 (51.26–84.37)	68.04 (54.45–89.20)	0.16^a^
Height, median (IQR), m	1.70 (1.56–1.80)	1.70 (1.56–1.78)	0.85^a^
BMI, median (IQR), kg/m^2^	23.69 (19.79–27.36)	25.15 (20.83–29.02)	0.17^a^
BSA, median (IQR), m^2^	1.84 (1.50–2.03)	1.79 (1.58–2.03)	0.40^a^
Imaging Modality			
MRI, No. (%)	6 (24%)	1 (4%)	0.10^b^
CT, No. (%)	19 (76%)	24 (96%)	0.10^b^
Reason For Imaging Scan			
Abdominal Pain, No. (%)	8 (32%)	7 (28%)	
Trauma, No. (%)	4 (16%)	4 (16%)	
Cancer, No. (%)	5 (20%)	3 (12%)	
Pancreatitis, No. (%)	4 (16%)	0 (0%)	
Crohn’s Disease, No. (%)	0 (0%)	3 (12%)	

Abbreviations: IQR, interquartile range; BMI, body mass index; BSA, body surface area; MRI, magnetic resonance imaging; CT, computed tomography

^a^ P value derived using Wilcoxon Signed-Rank Test

^b^ P value derived using Fisher’s Exact Test

### Pancreas Volume is Smaller in Patients with Type 1 Diabetes

**Fig 2:**
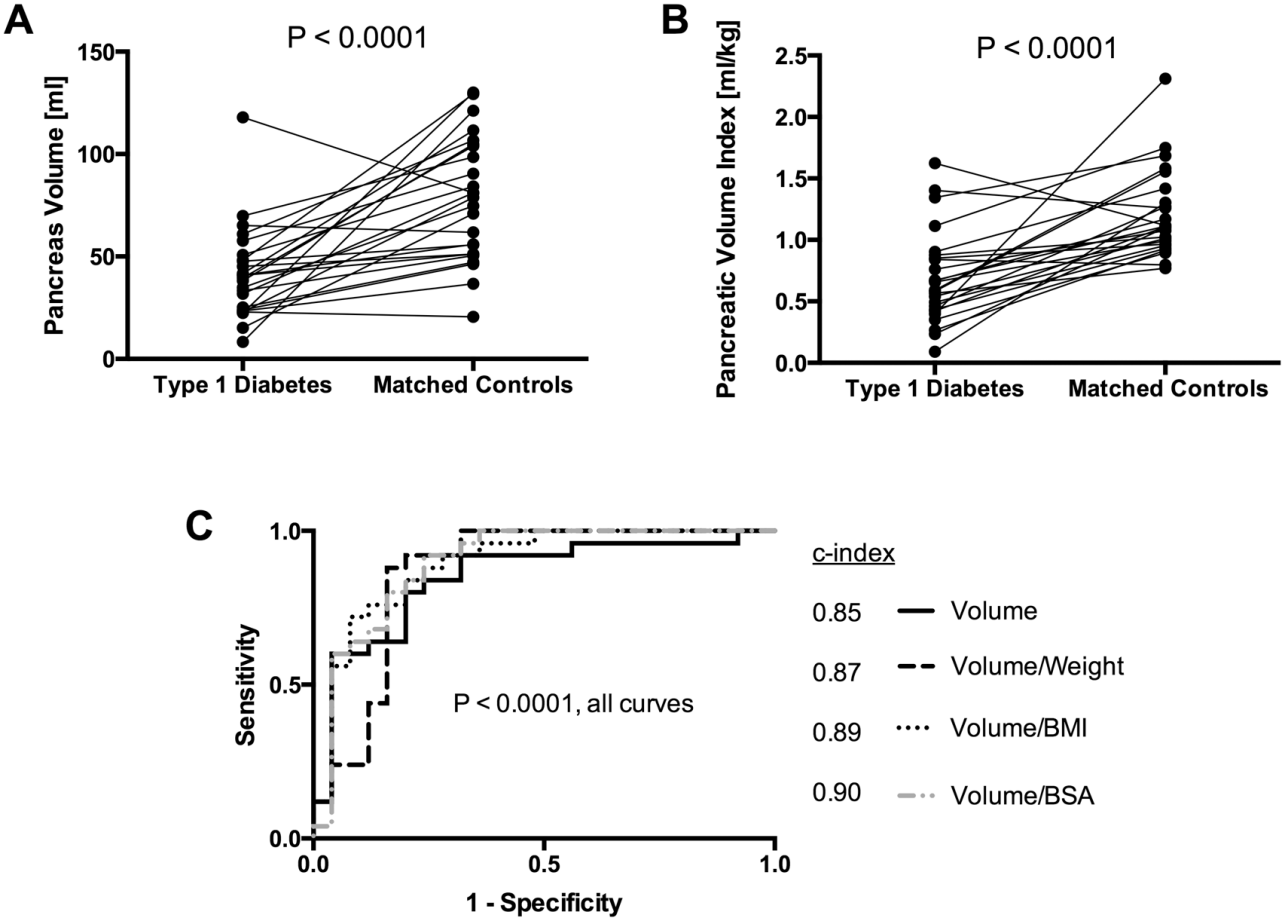
Pancreas volume is smaller in patients with type 1 diabetes than sex-, age-, and weight-matched controls. A) Pancreas volume, as determined from medical images, is smaller in patients with type 1 diabetes than matched controls (P < 0.0001). B) Pancreatic volume index, derived by dividing pancreas volume by subject body weight, is smaller in patients with type 1 diabetes than matched controls (P < 0.0001). C) Receiver operating characteristic curve indicates that pancreas volume and pancreas volume normalized by body weight, BMI, and BSA all discriminate patients with type 1 diabetes from matched controls (P < 0.0001). All four metrics provide similar diagnostic accuracy based upon c-index measurements.

The pancreas volume was 47% smaller (95% CI, 22% to 66%) in patients than in controls matched for sex, age, and weight (Fig 2A; average volume in patients 41.14 ± 22.35 ml; average volume in controls 77.77 ± 30.03 ml). To control for subject size, we calculated the pancreatic volume index by dividing the pancreas volume by the subject body weight [7]. This pancreatic volume index was 43% smaller (95% CI, 27% to 59%) in patients with T1D than matched controls (Fig 2B; average volume index in patients 0.69 ± 0.37 ml/kg; average volume index in controls 1.20 ± 0.35 ml/kg). To determine the best metric to normalize pancreas measurements for subject size, we divided pancreas volume by body weight, body mass index (BMI), and body surface area (BSA). Pancreas volume, volume normalized by body weight, volume normalized by BMI, and volume normalized by BSA all displayed significantly lower values in patients with T1D versus matched controls (P < 0.0001, pairwise comparisons between patients and controls). Pancreas volume was smaller in patients with T1D in 23 of 25 matched pairs when pancreas volume was normalized by BMI and in 22 of 25 matched pairs for the other three parameters. Receiver operating characteristic curves were generated to identify the diagnostic sensitivity and specificity for pancreas volume and volume normalized by body weight, BMI, and BSA (Fig 2C). All four parameters exhibited diagnostic utility (P < 0.0001, all parameters). The c-index values were not statistically different for pancreas volume (0.85, 95% CI 0.74 to 0.96), pancreas volume divided by body weight (0.87, 95% CI 0.76 to 0.98), pancreas volume divided by BMI (0.89, 95% CI 0.80 to 0.99), or pancreas volume divided by BSA (0.90, 95% CI 0.80 to 0.99).

### Pancreas Volume Declines with Disease Duration

**Fig 3:**
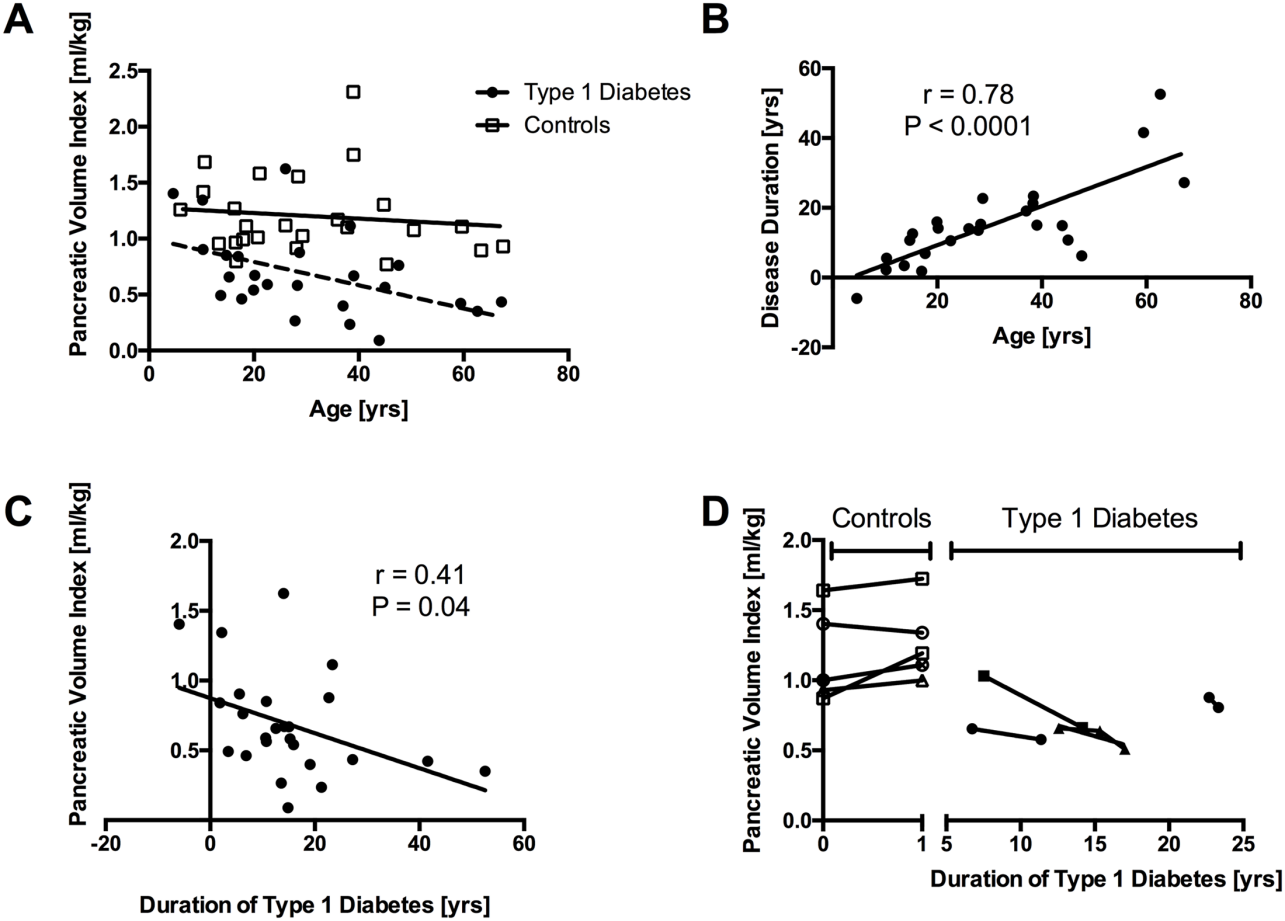
Pancreas volume is smaller with longer duration of type 1 diabetes. A) Pancreatic volume index is similar across the age range analyzed in control subjects (P = 0.56), but is smaller with age in patients with type 1 diabetes (P = 0.02). B) Patient age at the imaging scan correlates linearly with the duration of type 1 diabetes (P < 0.0001). C) Pancreatic volume index is smaller with increasing duration of type 1 diabetes (P = 0.04). D) In individual patients receiving multiple longitudinal imaging scans (N = 4) pancreatic volume index declines monotonically, whereas controls with repeated imaging scans do not display a decline in pancreas volume (P = 0.03).

Pancreas volume measurements were made in a wide age range (4–67 years old, patients; 5–67 years old, controls). Pancreatic volume index was similar across the range of ages examined in controls (Fig 3A; P = 0.56). In contrast, in patients with type 1 diabetes, pancreatic volume index was lower with increasing age (Fig 3B; P = 0.02). Patient age at the imaging time point correlated linearly with duration of type 1 diabetes at that time point (Fig 3B; r = 0.78, P < 0.0001). Pancreatic volume index in patients with T1D was lower with increasing disease duration (Fig 3C; P = 0.04). The average decline in pancreatic volume index was 0.013 ml/kg per year (95% CI 0.24 to 0.00064 ml/kg). Of note, one patient received a medical imaging study five years prior to diagnosis with T1D and had the second highest pancreatic volume index among patient scans. Additionally, in four of the patients with T1D multiple medical images separated temporally by one to seven years were available to assess pancreas volume. In these four patients, longitudinal measurements displayed a decline in pancreatic volume index of approximately 6% per year (Fig 3D). In five controls imaged at one year intervals, four subjects displayed a slight increase in pancreas volume index, while a fifth subject exhibited a small decline in pancreatic volume index (P = 0.03 slope of volume index over time versus control; Fig 3D).

### Pancreas Volume is Isotropically Smaller

**Fig 4:**
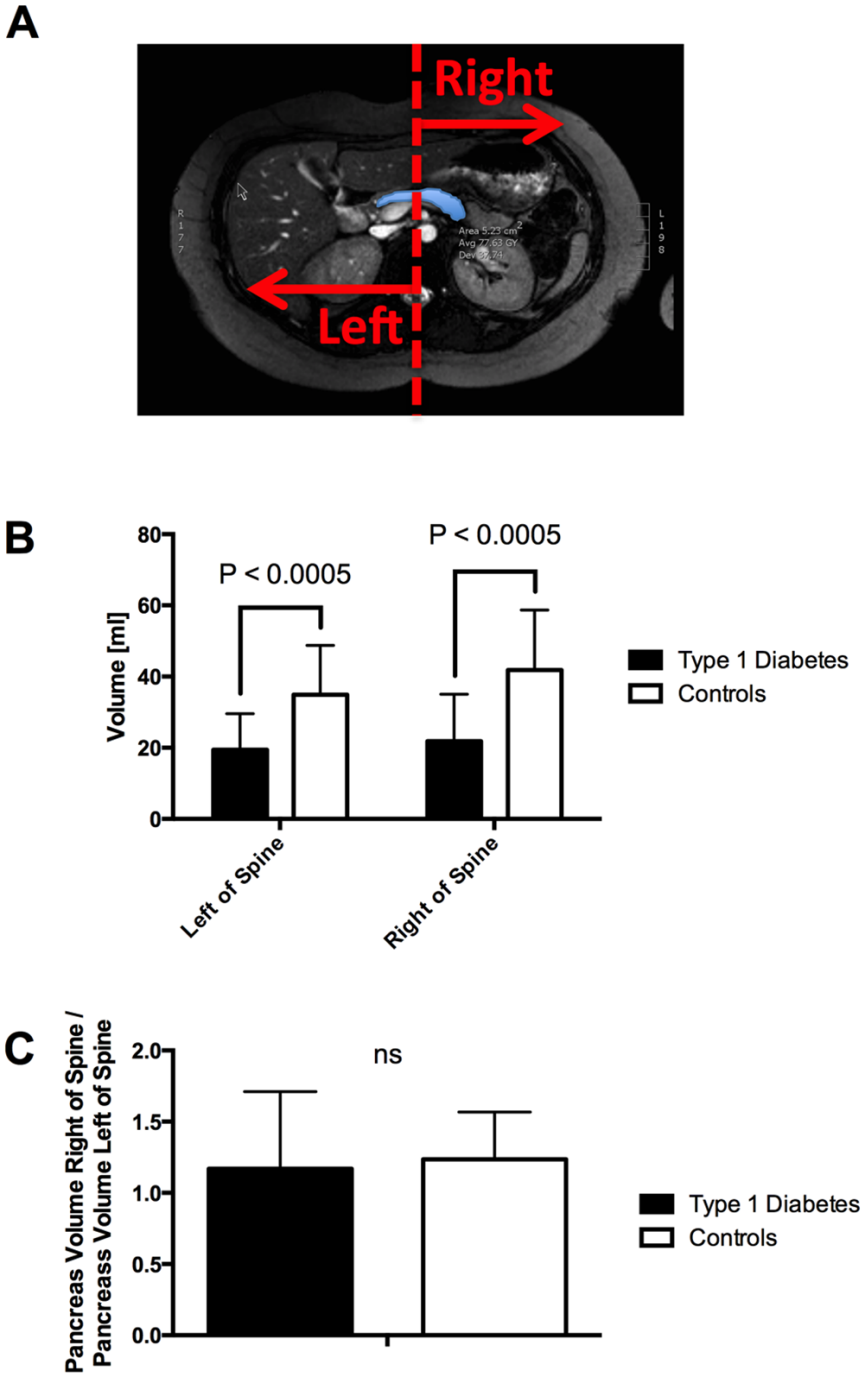
The pancreas volume in type 1 diabetes is reduced in both the right and left half of the abdomen. A) The abdomen was bisected by a midsagittal plane from spine to navel and pancreas volume was calculated for each half of the abdomen. B) The pancreas volume to the left of the spine and right of the spine were both smaller in patients with type 1 diabetes (P < 0.0005). C) The ratio of the pancreas volume to the left and right of the spine was similar in patients with type 1 diabetes and controls.

To determine whether the entire pancreas is smaller in type 1 diabetes or whether solely the pancreas head or tail is smaller, we bisected the abdomen into left and right halves using a midsagittal line drawn from spine to navel (Fig 4A). Pancreas volume to the radiological left of the spine (encompassing the pancreas head and part of the pancreas body) was smaller in patients with T1D (P < 0.0005; Fig 4B; average volume left of spine in patients 19.43 ml; average volume left of spine in controls 34.88 ml). Pancreas volume to the radiological right of the spine (including the pancreas tail and part of the pancreas body) was also smaller in patients with T1D (P < 0.0005; Fig 4B; average volume left of spine in patients 21.82 ml; average volume left of spine in controls 41.81 ml). However, the ratio between the pancreas volume right and left of the spine was similar in patients with T1D and controls (Fig 4C; average volume ratio in patients 1.17 ± 0.54; average volume ratio in controls 1.24 ± 0.33).

### Correlation of Pancreas Volume and Other Metrics

In patients with type 1 diabetes, glycated hemoglogin (HbA1c) measurements suggested an association between increased pancreatic volume index and poor glycemic control, although this association did not reach statistical significance (P = 0.07, S1A Fig). Patients with an insulin pump had a lower pancreatic volume index compared with those receiving insulin injections (0.46 ± 0.28 ml/kg vs. 0.79 ± 0.34 ml/kg, P = 0.02, S1B Fig). This correlation between insulin delivery approach and pancreatic volume index was preserved when duration of T1D was included in multivariable regression (P = 0.045). However, this correlation was not significant when HbA1c was included in multivariable regression (P = 0.14). Reflecting this relationship, patients on insulin pumps in this study tended to have a lower HbA1c than those receiving insulin injection (S1C Fig). There was no correlation between HbA1c and duration of disease. There was also no correlation between pancreatic volume index and the imaging modality used, either MRI or CT (P = 0.38). We found no correlation between pancreatic volume index and sex (P = 0.57), previous pregnancy (P = 0.31), or the number of previous pregnancies (P = 0.15). Among patients with type 1 diabetes, we found no association between pancreatic volume index and presence of weight loss at diagnosis (P = 0.36), diabetic ketoacidosis (P = 0.41), or pancreatitis (P = 0.61). To ensure that the presence of T1D cases with pancreatitis and controls with Crohn’s disease did not affect interpretation of results, both sets of matched pairs were independently omitted and analysis was repeated. Pancreatic volume index was smaller in patients with T1D when cases with pancreatitis (P < 0.0001) or controls with Crohn’s disease (P < 0.0001) were omitted from analysis.

## Discussion

This retrospective study demonstrates the utility of well-documented and searchable electronic medical records to investigate pancreas size in patients with type 1 diabetes. The use of archived medical images from the electronic medical record is particularly cost effective given the high cost of medical imaging procedures. Using archived medical imaging studies we found that patients with type 1 diabetes have a smaller pancreas than matched controls. This reduction is dramatic: patients with type 1 diabetes had a pancreas that was nearly half the size of their matched counterparts. The magnitude of this decline in pancreas volume suggests exocrine involvement in type 1 diabetes and is in agreement with previous studies in long-standing T1D [16, 17]. Studies of pancreas volume in new-onset T1D have demonstrated a smaller magnitude of reduction [6, 7], suggesting that pancreas volume declines with increasing duration of T1D. However, there are currently conflicting reports on whether pancreas volume decreases with disease duration [2] or is not influenced by duration [18, 19]. Leveraging the wide range in duration of T1D included in this study (5 years before diagnosis to 52 years after diagnosis), we found that pancreas volume declined with longer disease duration. Additionally, four patients with T1D who received multiple imaging scans also displayed a decline in pancreas volume in measurements separated by one to seven years. Five controls imaged at one year intervals did not exhibit a decline in pancreas volume. This finding suggests progressive pancreas atrophy in individual patients with T1D, including patients with long-standing disease. However, future longitudinal studies of pancreas volume in patients with T1D are needed to see whether this conclusion can be replicated in larger cohorts.

In order to compare individuals who are not weight-matched, pancreas volume measurements are commonly normalized for subject body build. Previous studies have used either body surface area [6, 19, 20] or body weight [7] to normalize pancreas volume for subject body build, and have each dubbed this normalized metric the ‘pancreatic volume index’. Our study compared normalization of pancreas volume by body weight, BMI, and BSA and found that each metric provided similar diagnostic utility using ROC curve analysis. In control subjects, normalization by body weight gave a pancreatic volume index metric that was insensitive to age. During normal adolescent growth and development when pancreas volume is known to increase [10, 21], body weight also increased at a similar rate. Thus the pancreatic volume index employed in this study, in which pancreas volume is normalized for subject weight, is not confounded by subject weight. This normalization to an age-insensitive value avoids confounding arising from normal growth and development and is useful for assessing pancreas volume across pediatric and adult patients. An autopsy study similarly found that normalization of pancreas weight by subject weight led to an age-insensitive measure [18]. It is unclear if the pancreatic volume index would decline beginning in the 7^th^ decade of life, a period known to exhibit pancreatic atrophy [10], as this was outside the age range analyzed in this study.

The smaller pancreas size in T1D reflects a global decline in pancreas volume, in which the pancreas volume to the right and left of the midsagittal plane is smaller. A previous autopsy study found that the decline in pancreas volume in T1D was dependent on the region of the pancreas studied [22]. However, our study did not detect any spatial heterogeneity in pancreas volume when bisecting the pancreas through the midline. Other imaging studies have used different anatomical landmarks to divide the pancreas into the head, body and tail [23–25]. However, due to the fact that not all of these landmarks were identifiable in all scans analyzed for this study we opted to use the simple, but robust, bisection of the abdomen. Of note, there was large inter-individual variation in the ratio of the pancreas volumes on either side of the spine. This large variation may limit our ability to detect regional differences in pancreas volume in this study.

There was an unexpected, but statistically insignificant, association between pancreatic volume index and HbA1c. Surprisingly, patients with poor glycemic control was associated with a higher pancreatic volume index, although this relationship did not reach statistically significant values. Gaglia, et al. also noted a similar trend between HbA1c and pancreas volume in new-onset T1D [6]. The reason for this association is unknown, and paradoxically suggests that pancreas volume is largest in patients with poor glycemic control. Patients receiving insulin injections had a larger pancreatic volume index than those on insulin pumps. This relationship likely reflects lower HbA1c in patients receiving insulin pumps, as this association was not significant when HbA1c was included in multivariable analysis.

This study is subject to the bias and confounding inherent to retrospective study. Patients with T1D and controls received imaging scans for some medical indication unrelated to their diabetes status. However, the clinical indications for the imaging study were largely well balanced between the groups. For the indications that were unbalanced between patients and controls (pancreatitis and Crohn’s disease), excluding these pairs from analysis still resulted in significant differences in pancreas volume. The imaging scans were not standardized for this study and both MRI and CT scans were analyzed. We did not detect any difference between measurements made using MRI and CT in this study, however, and previous studies using MRI [7, 17] and CT [10, 19] yielded similar pancreas volume measurements in control subjects. The calculated pancreas volumes are subject to measurements errors stemming from differences in image resolution between scans as well as inter- and intra-observer variability. To avoid inter-observer variability in this study a single reader analyzed all images in this study. Subjects were not fasted before the imaging study, demonstrating that even in the absence of fasting imaging is still capable of detecting smaller pancreas volume in type 1 diabetes.

In conclusion, we found that pancreas volume is markedly reduced in patients with type 1 diabetes and that this diminution corresponds with disease duration. As the pancreatic islets constitute only 1–2% of the pancreas volume, the extent of pancreas volume loss suggests exocrine involvement in T1D and challenges the established paradigm of solely beta cell destruction. Potential causative factors include the loss of the tropic effects of insulin on the surrounding exocrine tissue [26] and immune-mediated insult to the exocrine pancreas [27]. Further mechanistic studies are needed to identify the cause of pancreas atrophy in T1D and determine the potential clinical relevance. Alterations to the exocrine pancreas may be amenable to medical imaging, and, in concert with pancreas volume measurements, may prove useful for early detection of T1D and evaluation of new therapeutics. A recent report indicating reduced pancreas size in patients with type 2 diabetes [28] suggests that MRI may also be useful in studies of type 2 diabetes. Importantly, the use of MRI to measure pancreas volume does not employ ionizing radiation and does not require administration of a contrast agent, and thus is suitable for longitudinal imaging of pediatric patients.

## Supporting Information

S1 Fig Pancreatic volume index is associated with poor glycemic control. A) Pancreatic volume index is higher with higher values of glycated hemoglobin, although this did not reach statistical significance (P = 0.07). B) Pancreatic volume index is higher in patients who receive insulin via injection than pump (P = 0.02). C) Patients receiving insulin injections (solid circles) have higher glycated hemoglobin at the point of this study than those who are on insulin pumps (open squares). The correlation between insulin delivery mechanism (pump vs. injection) was not seen when glycated hemoglobin is included in multivariable analysis (P = 0.14).
(TIFF)

S1 Table Information on each T1D patient’s medications, HbA1c, form of insulin therapy, and whether the patient had diabetic ketoacidosis. (DOCX)

## Abbreviations

T1D: Type 1 diabetes
MRI: magnetic resonance imaging
CT: computed tomography
ICD-9: International Classification of Diseases 9th edition codes
HbA1c: glycated hemoglobin
BMI: body mass index
BSA: body surface area
