# Supplementary material for: Use of the Electronic Medical Record to Assess Pancreas Size in Type 1 Diabetes
Source: PLoS One. 2016 Jul 8;11(7):e0158825. doi: 10.1371/journal.pone.0158825 (PMC4938534; doi:10.1371/journal.pone.0158825)
Supplement: S1 Table — (DOCX) [file pone.0158825.s002.docx]

| Record Number | Diabetic Ketoacidosis | Medications | HbA1c [%] | Form of Insulin Therapy |
| --- | --- | --- | --- | --- |
| 1 | NA | humalog, lasix, phenergan, famotidine, alprazolam, cyclobenzaprine, metoclopramide, gabapentin, NuvaRing, amitriptyline, anaspaz, maxalt, cymbalta, tylenol, aleve | NA | Pump |
| 2 | NA | novolog, asmanex, singulair, zyrtec, albuterol sulfate | 7.8 | Pump |
| 3 | Present | apidra insulin, lantus | 11.9 | Injection |
| 4 | NA | humalog, propylthiouracil | 7.4 | Pump |
| 5 | Present | insulin, miralax | NA | Injection |
| 6 | Present | lantus, novolog | 10 | Injection |
| 7 | Present | insulin, famotidine, morphine | 10.9 | Injection |
| 8 | Present | lantus, novolog, keppra | 13.8 | Injection |
| 9 | Present | promethazine, alprazolam, beano, align, levothyroxine, lantus, humalog | 9.5 | Injection |
| 10 | NA | lantus, novolog, epinephrine, kariva, | 6.3 | Injection |
| 11 | NA | lisinopril, simvastatin, novolog, omeprazole, fexofenadine, Vesicare, Depakote, Bupropion, Levoxyl, lyrica, cetaphil | 7.5 | Pump |
| 12 | NA | piperacillin-tazobactam inj, levofloxacin inj, heparin, zolpidem, levothyroxine, gabapentin, mirtazapine: remeron, buspirone: buspar, ondansetron injection: zofran, hydromorphone, advair, insulin, simvastatin, irbesartan, metoprolol: lopressor, percocet | 7.9 | Pump |
| 13 | NA | metoprolol, asprin, humalog, lipitor, ramipril, zetia, Venlafaxine, | 8.9 | Pump |
| 14 | NA | humalog | 10.1 | Pump |
| 15 | Present | lantus, novolog, Levothyroxine | 10.3 | Injection |
| 16 | NA | insulin, tramadol, hydrocortisone, exogenous corticosteroids | 7.8 | Pump |
| 17 | NA | novolog, lantus | 7.8 | Injection |
| 18 | NA | novolog, nexium, synthroid, lisinopril, promethazine | 8 | Pump |
| 19 | NA | toprol, lantus, humalog, cozaar, buspar, promethazine | 6.9 | Injection |
| 20 | Present | novolog, gabapertin, eletriptal, promethazine | 12.5 | Injection |
| 21 | NA | levemir, xanax, zetia, senokot, novolog, lantus, tacrolimus, azelastine | 6.4 | Injection |
| 22 | Present | promethazine inj: phenergan, clonazepam: klonopin, temazepam, insulin, enoxaparin, lortab, hydromorphone | 10.7 | Injection |
| 23 | Present | Simvastatin, linsinopril, NovoLog, calcium, iron, aspirin, Prevacid, acyclovir, Percocet, omeprazole, simvastatin, amitriptyline, oxycodone, vitamin B12, Humulin, Levemir, ferrous sulfate, tizanidine | 8.6 | Pump |
| 24 | Present | valium, skelaxin, insulin | 12.8 | Injection |
| 25 | Present | novolog, lantus | 9.4 | Injection |
